# Amazonia Seasons Have an Influence in the Composition of Bacterial Gut Microbiota of Mangrove Oysters (*Crassostrea gasar*)

**DOI:** 10.3389/fgene.2020.602608

**Published:** 2021-02-12

**Authors:** Marcos Vinícius Reis Conceição, Sávio Souza Costa, Ana Paula Schaan, Ândrea Kely Campos Ribeiro-dos-Santos, Artur Silva, Diego Assis das Graças, Maria Paula Cruz Schneider, Rafael Azevedo Baraúna

**Affiliations:** ^1^Laboratory of Genomics and Bioinformatics, Center of Genomics and Systems Biology, Institute of Biological Sciences, Federal University of Pará, Belém, Brazil; ^2^Laboratory of Biological Engineering, Guamá Science and Technology Park, Belém, Brazil; ^3^Laboratory of Medical and Human Genetics, Institute of Biological Sciences, Federal University of Pará, Belém, Brazil

**Keywords:** mangrove oyster, oyster, oyster microbiota, Amazonia, *Crassostrea gasar*

## Abstract

The mangrove oysters (*Crassostrea gasar*) are molluscs native to the Amazonia region and their exploration and farming has increased considerably in recent years. These animals are farmed on beds built in the rivers of the Amazonia estuaries and, therefore, the composition of their microbiome should be directly influenced by environmental conditions. Our work aimed to evaluate the changes in bacterial composition of oyster's microbiota at two different seasons (rainy and dry). For this purpose, we amplified and sequenced the V3-V4 regions of the 16S rRNA gene. Sequencing was performed on the Illumina MiSeq platform. According to the rarefaction curve, the sampling effort was sufficient to describe the bacterial diversity in the samples. Alpha-diversity indexes showed that the bacterial microbiota of oysters is richer during the rainy season. This richness is possibly associated with the diversity at lower taxonomic levels, since the relative abundance of bacterial phyla in the two seasons remained relatively constant. The main phyla found include Firmicutes, Bacteroidetes, Actinobacteria, and Proteobacteria. Similar results were found for the species *Crassostrea gigas, Crassostrea sikamea*, and *Crassostrea corteziensis*. Beta-diversity analysis showed that the bacterial composition of oyster's gut microbiota was quite different in the two seasons. Our data demonstrate the close relationship between the environment and the microbiome of these molluscs, reinforcing the need for conservation and sustainable management of estuaries in the Amazonia.

## Introduction

The phylum Mollusca is one of the largest and most important in the animal kingdom. From the six classes that make up the phylum, we can highlight the Bivalvia class, composed of about 7,500 species of soft-bodied animals protected by a shell, which acts as a skeleton for the connection of muscles and protects against predators (Gosling, 2003; Dame, 2011). Oysters are molluscs belonging to the *Ostreidae* family found in various marine and estuarine environments around the globe (Dame, 2011). These animals breathe and feed using their gills, having a food base of microalgae, phytoplankton, microzooplankton, dissolved organic matter, and bacteria (Gosling, 2003).

As filter feeding animals, oysters tend to accumulate a large number of microorganisms present in the water, which can compose their gut microbiota (Harris, 1993; Li et al., 2017). The environment has a fundamental and highly restrictive role in the composition of the gut microbiota of these animals; in addition to that, different species developing in the same environment may have different bacterial populations composing their microbiota due to a difference in the filtration capacity (Trabal et al., 2012; Clerissi et al., 2020). It has been reported that ingested bacteria increase in number in the intestines of some invertebrates. Moreover, the proliferation in the intestine can contribute to digestion, preconditioning the food, thus complementing the enzymes present in the digestive tract (Harris, 1993).

Some phyla have already been identified composing the microbiota of different species of oysters belonging to the *Crassostrea* genus (King et al., 2012; Chauhan et al., 2014; Fernández et al., 2014; Ossai et al., 2017; Vezzulli et al., 2018). The phylum Proteobacteria is the most abundant, followed by Firmicutes and Bacteroidetes, additionally several other bacterial taxa have already been described composing oysters gut microbiota (Li et al., 2017). The microbiota of marine oyster species such as *Crassostrea gigas* is predominantly composed by marine bacterial genera such as *Neptuniibacter, Marinicella, Rhodovulum*, and *Oceanicola* (Fernández et al., 2014). In addition, cultivation site and the oyster's life cycle influence the composition of its microbiota (Fernández et al., 2014). The genera *Pseudoalteromonas* and *Vibrio* were the most abundant in the same *C. gigas* cultivated in the northwestern Italy (Vezzulli et al., 2018).

Prior to the beginning of this research, only studies using classical microbiology methods to assess the gut microbiota of *Crassostrea gasar* oysters were available (Amadi, 2016; Oliveira et al., 2020). *C. gasar* is a synonymous species to *C. brasiliana*, which is found in estuaries along the Brazilian coast (Varela et al., 2007; Melo et al., 2010). This species is vastly used in oyster culture sites in the north of the country for its better tolerance to salinity variation and adaptability to higher water temperature (Funo et al., 2018). Considering that the production and consumption of oysters, as well as other molluscs, has grown expressively in the last 8 years in the northern region of Brazil, assessing these oysters' gut microbiota is important not only for the aquaculture population and consumers but also for the scientific community, given that these oysters have never been studied to such level. The results obtained in this research allowed us to assess the bacterial gut microbiota of *C. gasar* oysters in two different seasons of the year.

## Method

### Data Collection

The sampling took place in two periods, one in the dry season (T1) on October 3rd, 2018 and the other in the rainy season (T2) on April 25th, 2019. Twenty-four oysters were collected, 12 in each period, from a cultivation site during the high tide in the village of Lauro Sodré (0.844866 S 47.884904 W) in Curuçá, in the northeastern region of the state of Pará, Brazil. In this study, two biological triplicates were used, one triplicate for each period. Each replicate had the gastrointestinal content from four oysters giving a final average weight of 10 g. Oysters were kept in a container with ice until being processed on the same day at the Laboratory of Genomics and Bioinformatics of the Federal University of Pará. The oysters were opened with sterile knives in sterile environment and the stomach and intestines were excised with the aid of a scalpel and forceps and preserved in tubes containing RNAlater 1:1 (m/v) (Invitrogen).

### Physicochemical Water Analysis

One liter of water was collected in triplicate at the same site, day and time of the oyster's collection. The water samples were collected in 1 L polypropylene bottles just above the oyster beds, packed in an isothermal box with ice and sent to the laboratory of the College of Sanitary and Environmental Engineering at the Federal University of Pará. Sampling and analytical methods were performed according to the procedures and recommendations described in the Standard Methods for the Examination of Water and Wastewater (Rice et al., 2012). Physicochemical parameters such as pH, salinity, total suspended solids, turbidity, and apparent color were analyzed by spectrophotometer or multiparametric probe (556 MPS; YSI, USA). Data were compared using one-way ANOVA and Tukey test (*p* < 0.01) in the program BioEstat v.5.0.

### DNA Extraction and Amplification

The DNeasy PowerSoil kit (Qiagen) was used for DNA extraction following the manufacturer's protocol. The concentration and purity of the extracted DNA was assessed using the NanoDrop micro volume spectrophotometer (Thermo Fisher Scientific), all samples showed an absorbance ratio at 260 and 280 nm ≥1.8. The integrity of the extracted genomic DNA was verified by electrophoresis on 1% agarose gel. The DNA was amplified by PCR using primers (Forward 5′ TCGTC-GGCAGCGTCAGATGTGT-ATAAGAGACAGCCTACGGGNGGCWGCAG and reverse 5′ GTCTCGTGGG-CTCGGAGATGTGT-ATAAGAGACAGGACTACHV-GGGTATCTAATCC) for the variable regions V3 and V4 of the 16S rRNA gene. The reactions were performed in tubes with 25 μL of total volume, denatured for 3 min at 95°C followed by 35 cycles with temperatures of denaturation, annealing and amplification of 95, 55, and 72°C, respectively, ending with 5 min of amplification at 72°C.

### Sequencing and Bioinformatics Analysis

Sequencing library was prepared using the Nextera XT Illumina kit, where sequencing adapters and barcodes were added to the samples. The sequencing was carried out at the Laboratory of Medical and Human Genetics (LGHM) at the Federal University of Pará through the Illumina MiSeq platform. PEAR v.0.9.8 (Zhang et al., 2014) was used to merge the reads. Primers and ambiguous nucleotides were subsequently removed and reads <225 bp were removed as well as reads with total expected error > 0.5. Sequences were clustered into Operational Taxonomic Units (OTUs) based on a dissimilarity of 3% and chimera sequences were removed with UPARSE pipeline (Edgar, 2013). Taxonomic analysis was performed with USEARCH v.11 comparing with the 16S rRNA database from SILVA v.1.3.2 (Edgar, 2010; Glockner et al., 2017). Functional properties of the microbial communities were predicted using iVikodak (Nagpal et al., 2019).

Statistical analysis was performed with Phyloseq, Vegan, and ggplot packages implemented in R Studio v.1.0.136 (McMurdie and Holmes, 2013). The following alpha-diversity indexes were determined: rarefaction curve, Shannon, Chao1, ACE, and Simpson. Non-Metric Multidimensional Scaling (NMDS) plot was used to compare the bacterial composition of the different seasons.

## Results

The data shown on Table 1 corresponds to the results of the physicochemical analysis for T1 (dry season) and T2 (rainy season). It was possible to notice a considerable variation in total suspended solids, turbidity, apparent color, conductivity, ammonium, total phosphorous, salinity, and precipitation. Such abiotic variations possibly had influenced in the gastrointestinal microbiota composition of oysters from T1 to T2. A low precipitation index (>2 mm) is expected during the Amazonia summer (T1) (Table 1).

**Table 1:** Physicochemical parameters.

**Parameter**	**T1**	**T2**	**Fold-change^**a**^**
Salinity (ppt)	9.6 ± 1.4^c^	4.43 ± 0.6^c^	−2.17
Precipitation (mm)	≤ 2	5.1–10	–
pH	6.6 ± 0.2	6.36 ± 0.15	–
Total suspended solids (mg L^−1^)	27.66 ± 3.78^c^	172.66 ± 11.01^c^	6.24
Turbidity (UNT)	4.56 ± 0.41^c^	162.66 ± 30.28^c^	35.67
Apparent color (UC)	10.73 ± 0.66^c^	241 ± 33.06^c^	22.46
Conductivity (μS cm^−1^)	247.33 ± 8.5^c^	369 ± 16.52^c^	1.49
Total phosphorous (mg L^−1^)	1.36 ± 0.15^b, c^	0.34 ± 0.12^b, c^	−4.0
Ammonium (mg L^−1^)	1.03 ± 0.15^b, c^	1.9 ± 0.05^b, c^	1.84
Nitrate (mg L^−1^)	1.76 ± 0.2^b^	1.23 ± 0.05^b^	−1.43
Nitrite (mg L^−1^)	0.007 ± 0.002	0.003 ± 0.001	–
Total nitrogen (mg L^−1^)	3.23 ± 0.3	2.4 ± 0.1	–

a *Fold-change was calculated by the ratio of the highest value to the lowest. A negative value indicates a decrease in that parameter in the rainy season (T2)*.

b *Values that had not complied with the Brazilian legislation [Conselho Nacional do Meio Ambiente (CONAMA), 2005]*.

c *Statistically significant values (p < 0.01)*.

*The values are the arithmetic mean and standard deviation of the triplicate for each sample*.

**Figure 1:**
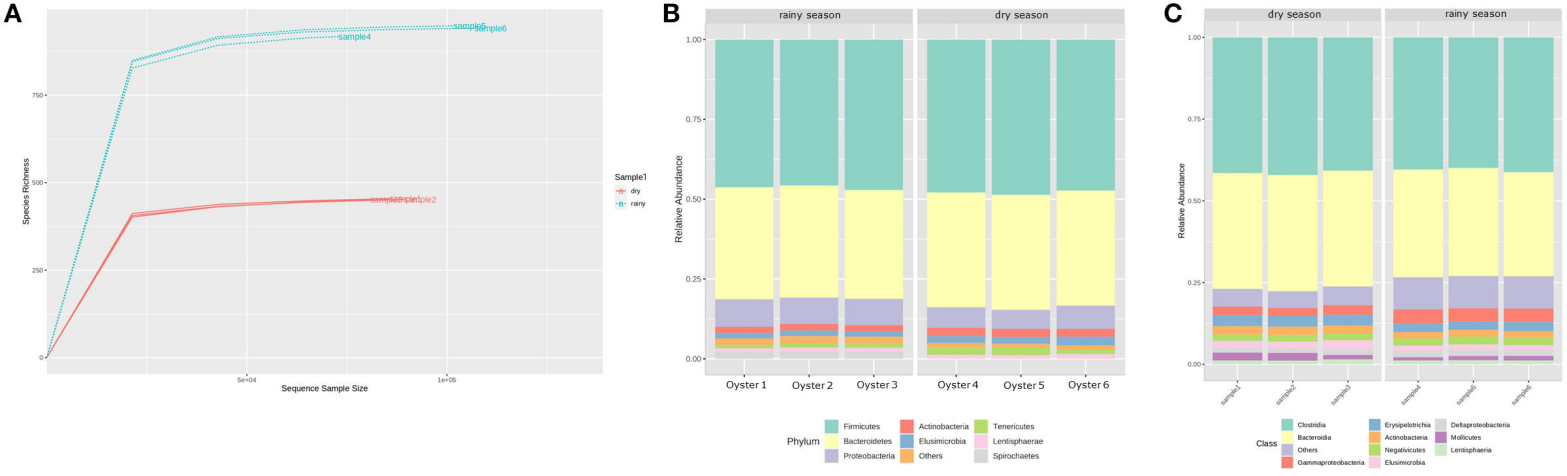
**(A)** Rarefaction curve of estimated OTUs in the 16S rDNA libraries. Samples from the rainy season (blue dotted lines) are notoriously richer compared to the dry season (red dotted lines). **(B)** Histogram of bacterial phyla diversity found in each replicate sequenced. Firmicutes, Bacteroidetes, Proteobacteria, and Actinobacteria were the most abundant phyla. No considerable difference can be noted in the relative abundance of these phyla. **(C)** Histogram of bacterial classes.

**Figure 2:**
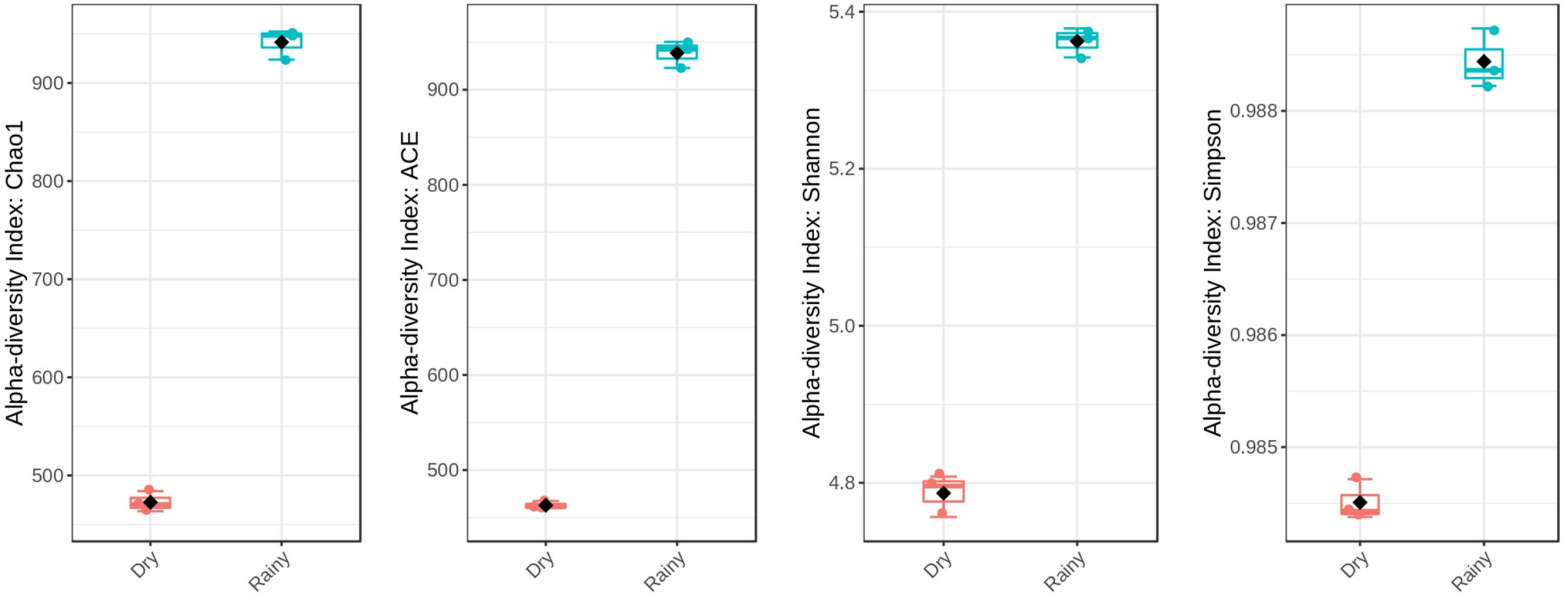
Alpha-diversity indexes comparing the two seasons analyzed. In all cases, the rainy season samples showed higher levels of diversity.

After sequencing and quality filter, each replicate presented 74,234–108,862 reads with an average size of 300 bp, totalling 163 Mbp of genetic information. According to the rarefaction curve, the sequencing effort was sufficient to describe the bacterial diversity of the samples (Figure 1A). In addition, rarefaction curve shows that the oyster microbiota is richer during the rainy season (Figure 1A), with values close to those of the respective diversity estimators, showing that the depth of the sequencing was sufficient for the description of the bacterial community (Figure 2). The most abundant phyla in the two periods analyzed were Firmicutes, Bacteroidetes, Proteobacteria, and Actinobacteria, in that order (Figure 1B). The recently characterized phylum Elusimicrobia was also found in significant abundance, followed by Tenericutes and Lentisphaerae.

**Figure 3:**
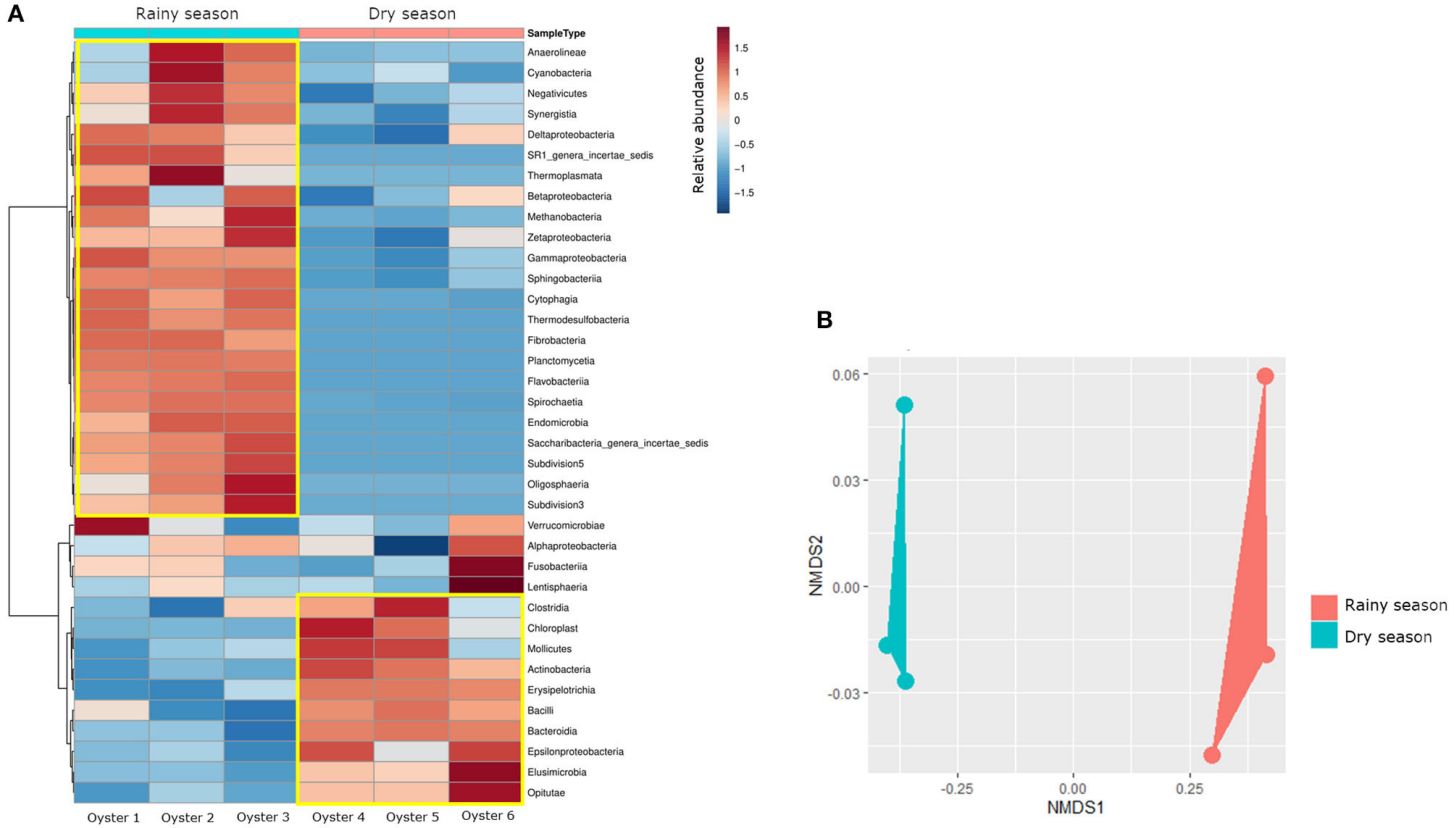
**(A)** Heatmap comparing the relative abundance of bacterial classes. The yellow square highlights the classes with higher relative abundance in each season. The dendogram on the left side reinforces the difference in the composition of bacterial classes in each season. **(B)** Non-Metric Multidimensional Scaling (NMDS) plot comparing the bacterial composition of samples. The samples were grouped according to the Amazonia seasons.

Despite the similar composition in bacterial phyla and classes (Figures 1B,C), the *C. gasar* microbiota showed significantly higher alpha-diversity indexes (Chao, Ace, and Shannon index) during the rainy season (Figure 2), but similar dominances (Simpson index). This result indicates that the change in the bacterial diversity occurs at lower taxonomic levels, since the relative abundance of bacterial phyla in the two seasons remained relatively constant (Figure 1B). Indeed, the heatmap in Figure 3A shows that some bacterial classes are abundant only in the rainy season such as Gammaproteobacteria, Betaproteobacteria, Zetaproteobacteria, Thermodesulfobacteria, Flavobacteria, Endomicrobia, among others. These differences are even more pronounced at the family level (Supplementary Figure 1). Epsilonproteobacteria was the most abundant Proteobacteria class during the dry season (Figure 3A). At the genus level, several taxa commonly associated with the gut microbiota of animals were found enriched in the oysters' gut during the dry season such as *Escherichia, Shigella, Bifidobacterium, Lactobacillus, Prevotella*, and *Ruminococcus* (Supplementary Figure 2). Flavonoid-degrading bacteria such as *Flavonifractor* were abundant during the rainy season (Supplementary Figure 2) (Ulbrich et al., 2015). As expected, some environmental genera were also found in the oysters' gut microbiota such as *Telmatospirilum* and *Desulfonatrobacter* (rainy season), and *Magnetococcus* and *Anaerosalibacter* (dry season). Comparing samples using Non-Metric Multidimensional Scaling (NMDS) plot it was evident that the bacterial diversity of oysters' gut microbiome was different in the two Amazonia seasons (Figure 3B).

Supplementary Figure 4 shows the abundant functions of the microbial communities predicted by iVikodak. Basic functional properties such as carbohydrate and amino acid metabolism, and DNA replication and repair were most abundant during rainy season. An important biochemical pathway for the host-microbiome interaction such as the metabolism of cofactors and vitamins was among the most abundant in the samples (Supplementary Figure 4).

## Discussion

During the dry season the sampling site presented a high salinity and low amount of total suspended solids (Table 1). In both periods, samples were considered as brackish water according to the Brazilian legislation [(Conselho Nacional do Meio Ambiente (CONAMA), 2005)]. Physicochemical parameters that had not complied with the Brazilian legislation were total phosphorous, nitrate and ammonium. Nitrate concentration did not change significantly between seasons (Table 1). The concentration of ammonium however was higher during the rainy season, possibly due to the carrying of organic matter from the soil to the estuary by the rains. Ammonia is a common product of biological degradation. The high temperature of the region and the near neutral water pH found are important factors that favor biocalcification and shell formation (Waldbusser et al., 2011). Salinity is also an important factor in oyster farming. Sampling point showed 9.6 and 4.43 ppt in the dry and rainy seasons, respectively. These low salinity values can be explained by the distance from the ocean. The village of Lauro Sodré is the most distant of all oyster farming villages on the Amazonia coast. Interestingly, it has the largest natural nursery system of oyster seeds of the region (Sampaio et al., 2019).

Firmicutes, Bacteroidetes, Proteobacteria, and Actinobacteria were the most abundant phyla in the two seasons analyzed (Figure 1B). The same phyla were described as the most abundant in the microbiota of the Pacific oysters *Crassostrea sikamea* and *Crassostrea gigas*, and the Mexican oyster *Crassostrea corteziensis* (Fernández et al., 2014). However, unlike our findings, Fernández et al. (2014) observed Proteobacteria as most abundant phylum in the Pacific and Mexican oysters when analyzing only the resident microbiota. A deeper investigation about the resident and transient microbiota of mangrove oysters should be performed to better elucidate microbial dynamics in such environments.

Regarding other species, the bacterial microbiota of *Crassostrea viriginca* is mainly dominated by the phylum Cyanobacteria (Chauhan et al., 2014), for which in our analysis, the number of reads mapped to the Cyanobacteria phylum was insignificant. Interestingly, Chauhan et al. (2014) identified *Cyanobacteria* spp. as the dominant taxon in the water column of the *C. virginica* farming site, which indicates an intrinsic connection between the oyster microbiome and the aquatic environment. *C. virginica* grows in the salty waters of the Atlantic coast while *C. gasar* grows in the brackish waters of the Amazonia estuaries. The difference between these environments is probably an important factor that contributes to the difference between the microbiota of these two oyster species.

Beta-diversity analysis demonstrated that there is a change in the bacterial composition of the oyster microbiota comparing the dry and rainy seasons (Figure 3B). Zurel et al. (2011) also found significant seasonal variation in bacterial populations in the gill of Indo-Pacific oyster *Chama pacifica*. Other biotic and abiotic factors have already been described influencing the composition of bacterial microbiota of bivalve molluscs. Offret et al. (2020) showed that *C. gigas* modifies its bacterial microbiota according to the farming position in an intertidal zone. Bernal et al. (2016) showed that farming of *C. sikamea* with potentially probiotic bacterial genus, such as *Streptomyces*, leads to a change in the oyster's microbiota. In our data, relevant changes in terms of abundance were observed at the genus level (Supplementary Figure 2). The metabolism of carbohydrates, fatty acids, amino acids and even antibiotic resistance genes were more abundant in the rainy season (Supplementary Figures 4, 5), when the bacterial microbiota was richer (Figure 2). The inference of metabolism based on 16S sequencing has already been used to compare the gut microbial communities of human populations living in urban and tribal areas (Singh et al., 2019), landfill sites with different characteristics (Thakur et al., 2020), and polystyrene-degrading biofilms cultivated under different conditions (Tourova et al., 2020).

The sequencing of the 16S rRNA gene was previously used to assess bacterial diversity in *C. gasar* (Ostrensky et al., 2018). However, the authors evaluated the differences in the composition of bacterial microbiota of living oysters stored in two different conditions, and found that oysters stored exposed to the air at a temperature of 5–25°C have a less diverse bacterial microbiota and a smaller number of potentially pathogenic taxa (Ostrensky et al., 2018).

To the best of our knowledge, this is the first study to evaluate the bacterial microbiota of *C. gasar* farmed in their natural habitat. The sustainable management of mangrove oysters is extremely important for Amazonia. Our research is important for the comprehension of the relationship between the environment and the oyster's microbiome, which appears to be influenced by biotic and abiotic factors and may have an important influence in farming management and human consumption.

## Data Availability Statement

The datasets presented in this study can be found in online repositories. The names of the repository/repositories and accession number(s) can be found below: https://www.ncbi.nlm.nih.gov/genbank/, PRJNA661079.

## Author Contributions

MC and RB: collected the samples, analyzed and interpreted the data, and wrote the manuscript. SC and DG: contributed to the bioinformatics analyses. APS and ÂR-d-S: contributed to the sequencing analysis. AS, DG, MS, and RB: conceived the study. All authors contributed to the article and approved the submitted version.

## Conflict of Interest

The authors declare that the research was conducted in the absence of any commercial or financial relationships that could be construed as a potential conflict of interest.
