# Supplementary figures and images for: Amazonia Seasons Have an Influence in the Composition of Bacterial Gut Microbiota of Mangrove Oysters (Crassostrea gasar)
Source: Front Genet. 2021 Feb 12;11:602608. doi: 10.3389/fgene.2020.602608 (PMC7907636; doi:10.3389/fgene.2020.602608)

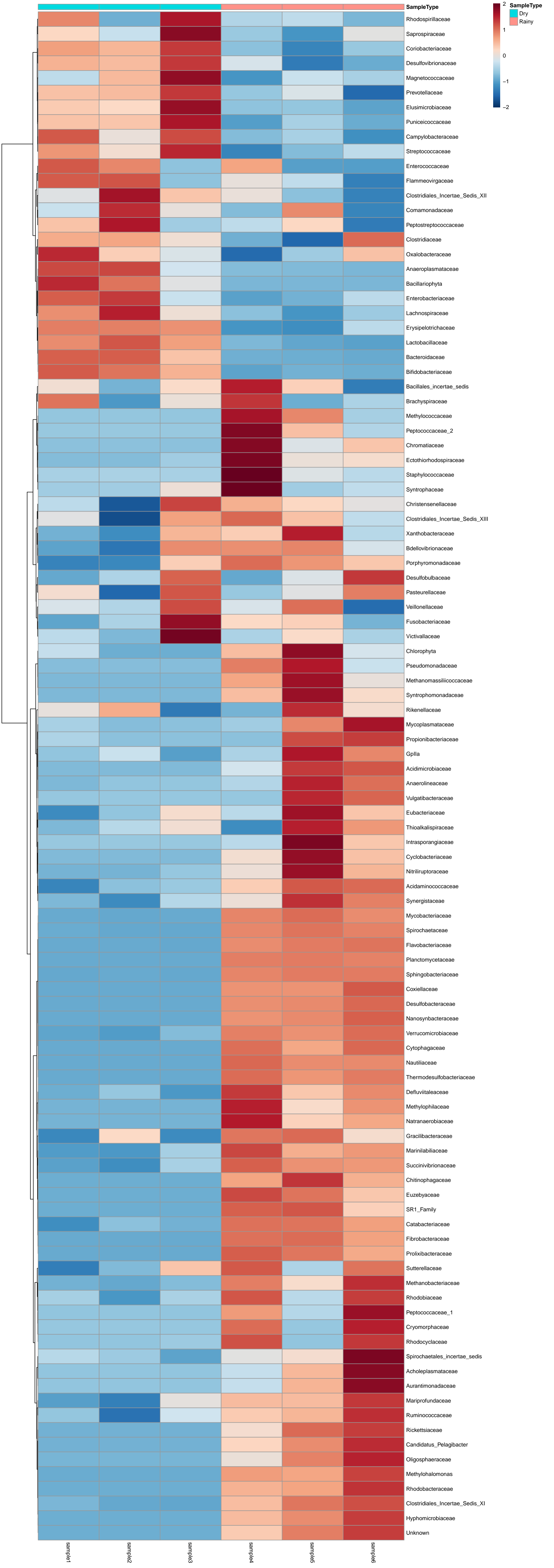

Supplement: Supplementary file 1 [file Data_Sheet_1.PDF]

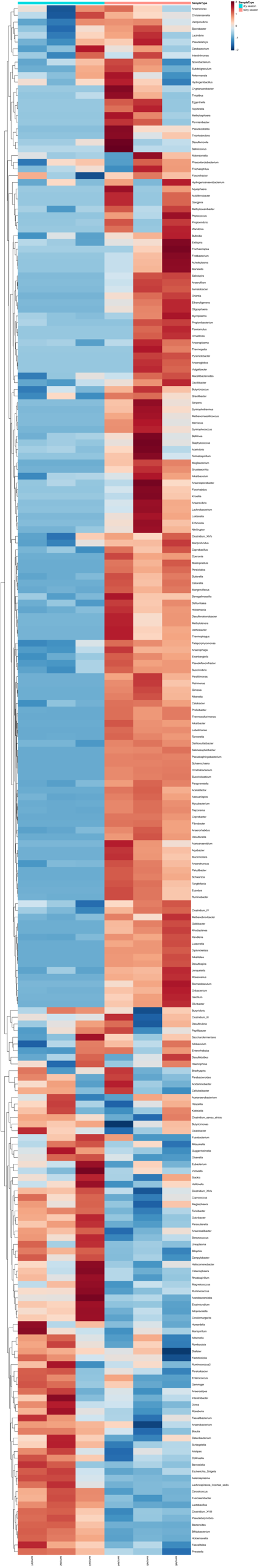

Supplement: Supplementary file 2 [file Data_Sheet_2.PDF]

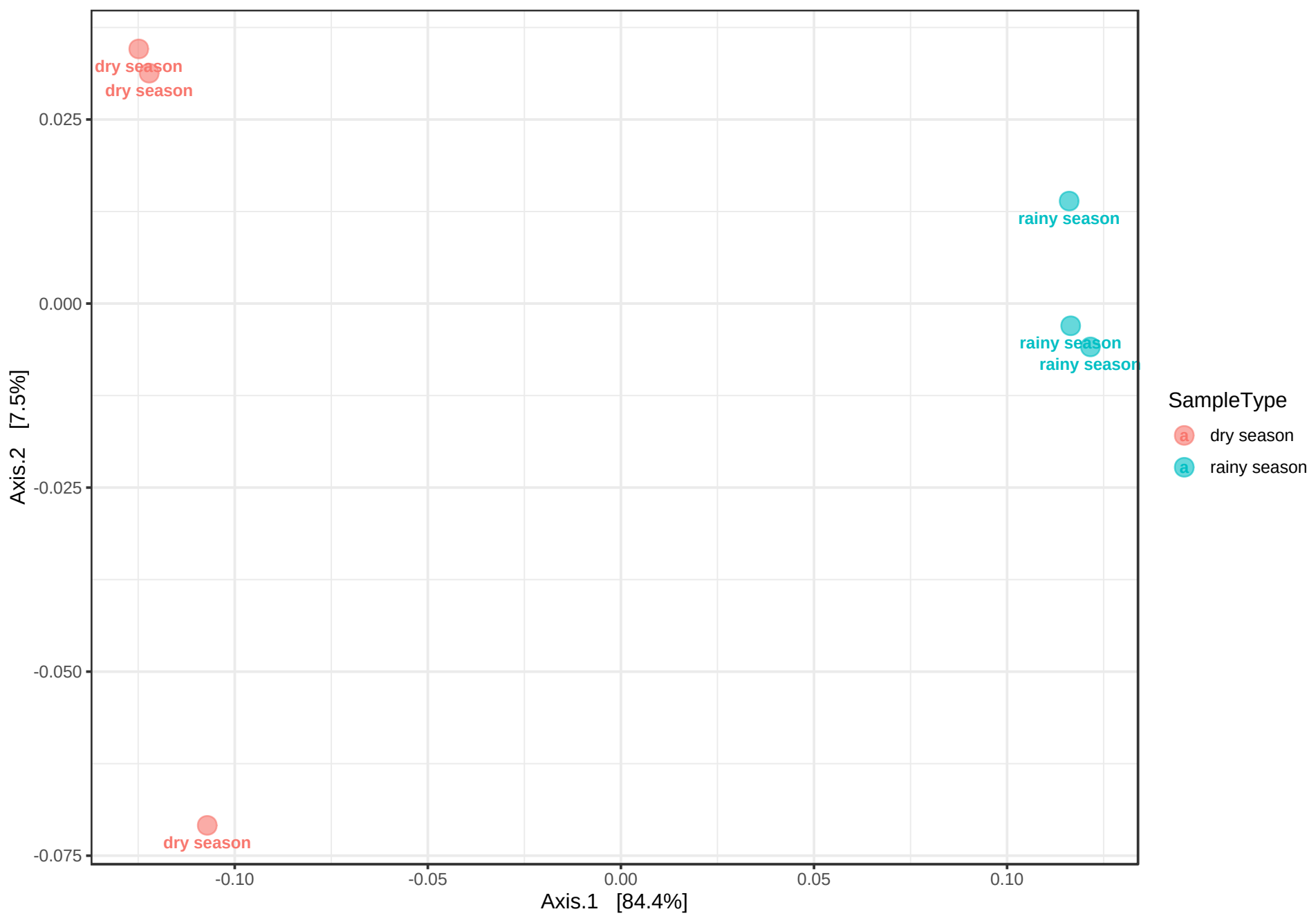

Supplement: Supplementary file 3 [file Data_Sheet_3.PDF]

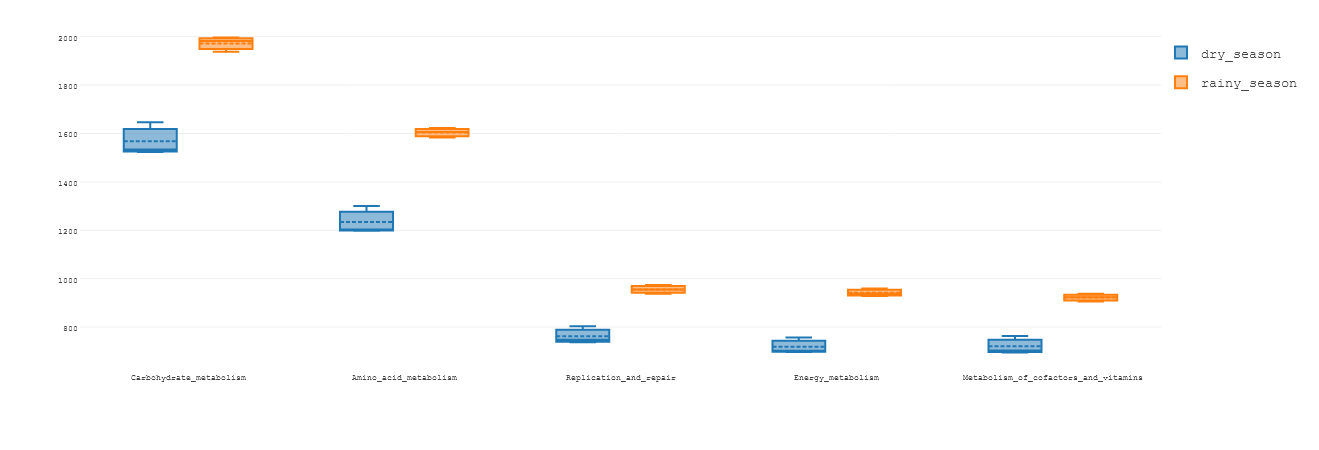

Supplement: Supplementary file 4 [file Image_1.PNG]

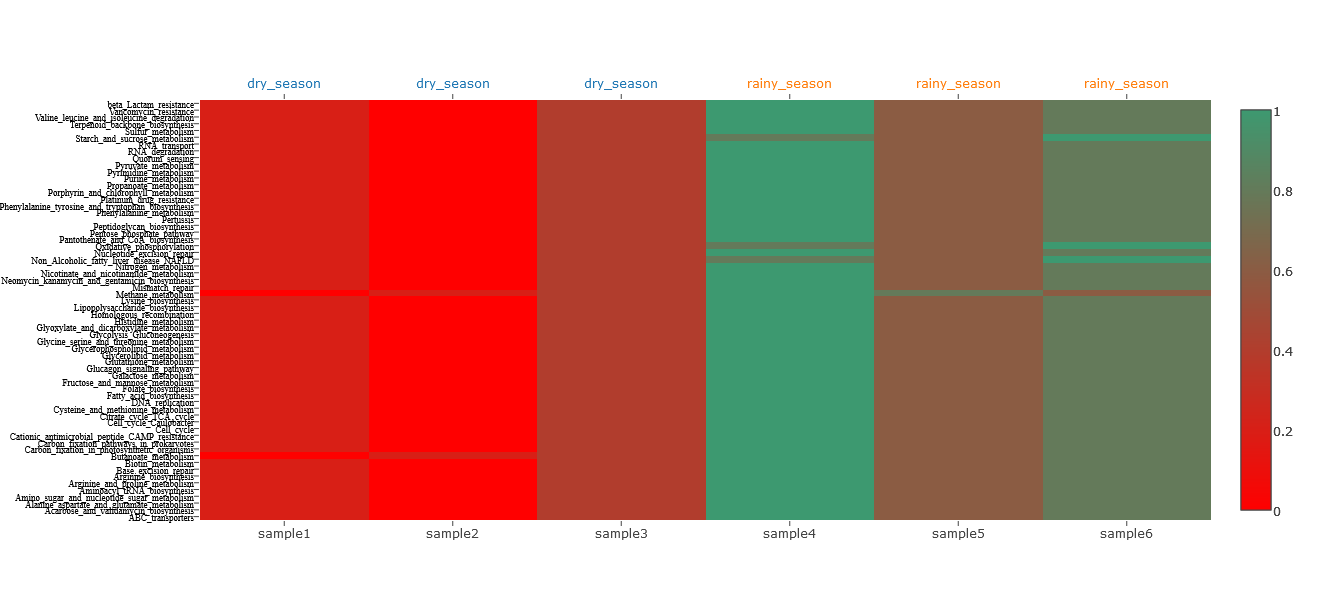

Supplement: Supplementary file 5 [file Image_2.PNG]
